# A semantic-based method for extracting concept definitions from scientific publications: evaluation in the autism phenotype domain

**DOI:** 10.1186/2041-1480-4-14

**Published:** 2013-08-12

**Authors:** Saeed Hassanpour, Martin J O’Connor, Amar K Das

**Affiliations:** 1 Stanford Center for Biomedical Informatics Research, Stanford, CA 94305, USA; 2 Geisel School of Medicine at Dartmouth, 46 Centerra Drive, Suite 330, Lebanon, NH 03766, USA

**Keywords:** Knowledge acquisition, Ontologies, Rules, Biomedical definitions, Autism phenotypes

## Abstract

**Background:**

A variety of informatics approaches have been developed that use information retrieval, NLP and text-mining techniques to identify biomedical concepts and relations within scientific publications or their sentences. These approaches have not typically addressed the challenge of extracting more complex knowledge such as biomedical definitions. In our efforts to facilitate knowledge acquisition of rule-based definitions of autism phenotypes, we have developed a novel semantic-based text-mining approach that can automatically identify such definitions within text.

**Results:**

Using an existing knowledge base of 156 autism phenotype definitions and an annotated corpus of 26 source articles containing such definitions, we evaluated and compared the average rank of correctly identified rule definition or corresponding rule template using both our semantic-based approach and a standard term-based approach. We examined three separate scenarios: (1) the snippet of text contained a definition already in the knowledge base; (2) the snippet contained an alternative definition for a concept in the knowledge base; and (3) the snippet contained a definition not in the knowledge base. Our semantic-based approach had a higher average rank than the term-based approach for each of the three scenarios (scenario 1: 3.8 vs. 5.0; scenario 2: 2.8 vs. 4.9; and scenario 3: 4.5 vs. 6.2), with each comparison significant at the p-value of 0.05 using the Wilcoxon signed-rank test.

**Conclusions:**

Our work shows that leveraging existing domain knowledge in the information extraction of biomedical definitions significantly improves the correct identification of such knowledge within sentences. Our method can thus help researchers rapidly acquire knowledge about biomedical definitions that are specified and evolving within an ever-growing corpus of scientific publications.

## Background

Biomedical knowledge is growing rapidly, and the majority of it is in an unstructured form in text. Researchers face a number of difficulties when trying to find relevant knowledge in this flood of information and to formalize it in a computational form for applications ranging from annotated publication repositories to automated decision support. In extracting relevant knowledge from scientific publications, the initial problem is finding articles relevant to a particular domain-related query. A secondary problem is to identify the portion of text within a retrieved article that contains relevant domain knowledge. Prior information extraction approaches in the biomedical domain, such as GoPubMed [1] and Textpresso [2], have shown that the use of pre-existing knowledge, encoded as class hierarchies, can address these two challenges. These past semantic-based methods, however, fall short in resolving a third problem: helping users identify specific instances of structured domain knowledge, not just the presence of biomedical concepts, within relevant text.

We have addressed this problem in our efforts to assist the information extraction needs of mental health experts who are developing a knowledge-based catalog of autism phenotypes [3]. Such phenotype concepts are represented as classes within a domain ontology and defined more precisely as rules expressing numeric or temporal cut-offs of measurements on standardized diagnostic tests [3]. The goal of our efforts is to provide an approach that can help researchers accurately identify existing phenotype definitions within text or facilitate the acquisition of knowledge about newly published phenotype definitions. Assisting clinical and genetics researchers acquire and maintain such rule-based classifications of phenotypes can facilitate their cataloging, comparison, and validation and ultimately enable the use of standardized biomedical definitions for robust, reproducible phenotype-genotype analyses.

In resolving this problem, we must address a number of challenges in information extraction, which we illustrate using following verbatim examples of phenotype definitions in two snippets taken from the same publication by Hus et al. [4].

Snippet Example 1

“One construct commonly used to stratify samples is age of language acquisition, based on age of first words or phrases. Delayed language is defined on the ADI-R by age of first words ≥ 24 and age of first phrases ≥ 33–36 months. …”

Snippet Example 2

“Language Acquisition Groups defined based on ADI-R items 9 (Age of First Words) and 10 (Age of First Phrases). Individuals were grouped as follows:

• NDW (not delayed words): acquired words ≤ 24 months

• DW (delayed words): acquired words > 24 months

• NW (no words): no words at time of ADI-R

• NDP (not delayed phrases): acquired phrases ≤ 33 months)

• DP (delayed phrases): acquired phrases > 33 months)

• NP (no phrases): no phrases at time of ADI-R)”

The first snippet appears in the introductory section of the article, whereas as the second snippet appears in the article’s Methods section. Both contain multiple definitions of autism phenotypes related to language acquisition, such as delayed words and delayed phrases, which are based on the autism diagnostic instrument, ADI-R. The intertwined definitions of multiple phenotypes within a single snippet indicate why methods using pattern-based concept recognition within a single sentence, such as used by Textpresso, or textual entailment in the form of a concept “is defined as…” would be limited in identifying such complex concept definitions.

**Figure 1:**
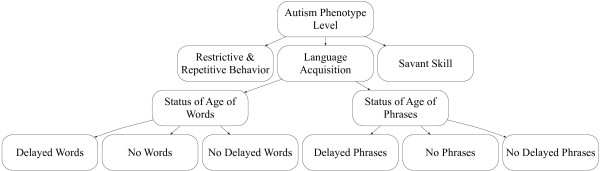
**A part of autism phenotype class hierarchy.** A portion of the class hierarchy showing the encoding of *Delayed Words* phenotype and other phenotype concepts defined in the two example snippets in the Background section.

In this paper, we have focused on the extraction of phenotype definitions from scientific articles and their acquisition as Semantic Web Rule Language (SWRL) [5] rule statements in pre-existing Web Ontology Language (OWL) [6] ontologies. In our work, autism phenotypes are categorized as an OWL class hierarchy and defined by a set of tests and measurements in SWRL rules. Considering the example of *Delayed Words* phenotype, which is defined in the above two text snippets, Figure 1 shows this phenotype and its relationship with other phenotypes in a small part of the autism ontology’s class hierarchy. As can be seen in this figure, *Delayed Words* phenotype is the direct descendent of *Status of Age of Words* and indirect descendent of *Language Acquisition* and *Autism Phenotype Level* concepts. Delayed Words phenotype SWRL rule definition specifies this phenotype according the snippet example 2 based on the criteria from definition ADI-R test. This rule indicates that if a child does not acquires the ability to speak words by the age of 24 months or earlier, then there is a delayed development in word acquisition and asserts this finding in the record of the subject who had that ADI-R questionnaire completed for him or her.

**Figure 2:**
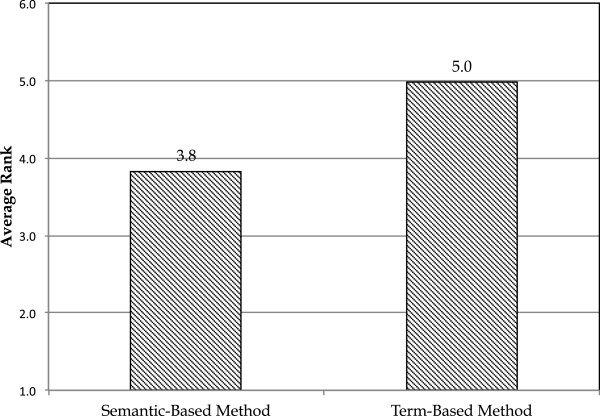
**Comparing semantic- and term-based methods in the first scenario.** Average ranks of the correct phenotypes found in a snippetfor the semantic- and term-based methods.

Delayed Words phenotype SWRL rule definition

ADI-R(?a) ^ adi-r2003:ADI_2003_acqorlossoflang_aword(?a, ?wordage) ^ swrlb:greaterThan(?wordage, 24) ^ adi_r2003:SubjectKey(?a, ?subjectID) ^ swrlx:createOWLThing(?phenotype, ?subjectID) → ‘Delayed word’(?phenotype) ^ autism-core:subject_has_quality_or_disposition(?subjectID, ?phenotype)

In prior work, we have presented semantic-based information retrieval that uses previously encoded knowledge about a domain, specifically a domain ontology and rule base, to identify papers relevant to phenotypes and to extract snippets of text most likely to contain their definitions [7,8]. In this paper, we present a novel semantic-based approach to identify which exact definition or related definition exists within a returned snippet of text in an article. Our approach must allow the knowledge base developer to handle three different scenarios in information extraction. In the *first scenario*, the snippet contains one or more rule-based definitions that are already encoded within the domain knowledge. In this situation, we provide the developer a set of concepts and their rules and allow the developer to simply associate the new text with the existing rule. In the *second scenario*, the concept exists with the domain ontology but the criteria used for defining that concept differ from that in the snippet. For example, there is a slight difference in the cut-off used in defining the concepts of delayed words and delayed phrases in the example snippets. We again provide the developer a set of concepts and their rules and permit the user to modify an existing rule to create a new alternative rule for that concept that matches the definition within the snippet. Finally, in the *third scenario*, the text contains a concept definition that has not been previously encoded within the domain knowledge. In this scenario, we present the developer a set of existing concepts that may be related, and assume that one of the existing rules for these concepts can be used as a template to acquire the rule for the new concept. Of note, all three of these scenarios may exist within a single snippet of relevant text.

In this paper, we show how we address the information extraction challenge of each of these three scenarios using a single vector-space modeling approach that incorporates terms and their weights based on previously encoded domain knowledge. In each scenario, we use the vector-space model to find the set of concepts and their rules that most closely match the biomedical definitions within the given text. We compare our semantic-based approach with a more conventional term-based mechanism and show that incorporating domain knowledge significantly improves the relevance of the ranked results. We have evaluated our framework by using it to extract autism phenotype definitions from text. Although we presented this work in the domain of autism phenotyping, our techniques do not depend on this particular domain, and are not restricted to OWL and SWRL frameworks. Our method is applicable to other domains and any structured knowledge format that consists of class hierarchies and rules.

### Related work

Efforts to automatically acquire rule-like knowledge have a long history in computer science and informatics research. The association rule-mining field, in particular, has developed an extensive array of such techniques [9]. The general aim is to discover significant relationships between variables in structured data and to encode these relationships as rules. The expansion of online information repositories has steered work towards extracting knowledge from less structured data sources. In the biomedical domain, the availability of a large number of abstracts and full-text publications from sources like PubMed has provided an impetus to the development of new techniques. Early work focused on automatically classifying the subjects of papers to help guide searches in particular domains. Many of these efforts have used ontologies in the key role of providing structured terminologies for this classification process, or indeed have extracted ontologies themselves [10-12]. In some cases, domain ontologies provide an initial controlled vocabulary for identifying terms.

More recent work has employed natural language processing techniques to infer relationships between the concepts described in a corpus. For example, Rinaldi et al. [13] described a method for extracting interactions between proteins from publications. Using related techniques, researchers have begun to attempt to automatically derive ontology hierarchies from text by extracting domain terms from a corpus and finding pairwise relationships between them [14-17]. Again, a variety of techniques are used to build these hierarchies: (1) documents are analyzed for syntactic patterns that indicate relationships between terms; (2) template-based approaches are used to describe syntactic patterns, which are then used to find relationships between terms; and (3) statistical methods are used to detect term co-occurrence, which can often indicate relationships.

In contrast, acquiring domain knowledge in the form of rules is more challenging because the logical relationships that can be modeled as rules can be significantly more complex [18]. Instead of simply detecting relationships between concepts pairs, the method must automatically link a series of these relationships together to build composite requirements, which can then be encoded as rules. Some work in rule acquisition used statistical methods to extract simple term-to-term entailment rules from text [19]. However such simple rules are very limited in modeling most typical domain knowledge. A more elaborate variant of this approach attempts to extract first-order Horn clauses from the text [20]. This method needs to be applied on a large corpus of text to gather statistical evidences to discover rules. Related efforts have attempted to extract first order logic rules by using machine learning methods [21]. These rules are learned from a set of positive and negative examples therefore are not suitable for use on free text. Also, rule editors such as SemEx [22], provide the functionality to annotate text with common business terms to help put together simple business rules in a semiautomatic manner.

Only a small number of approaches using ontology-based rule extraction methods are described in the literature. Duboue and McKeown described a system for capturing content selection rules from text that identify parts of a corpus that are relevant to a certain topic [23]. The system used a frame-based knowledge representation format to drive statistical methods to produce these types of rules from short segments of user-supplied text. This text is assumed to contain relevant rule-like information. Manine et al. [24] presented an approach for acquiring gene interaction rules from text, which were then encoded using ontologies. The approach used an existing ontology as input to an inductive logic algorithm, which used it to learn inference rules from pre-selected text. Park and Lee [25] developed an ontology-based method to extract rules semi-automatically from web documents. Like other rule acquisition approaches, the method required an existing domain ontology and manual selection of relevant web pages as method input. This approach used very basic WordNet-based NLP techniques, so was limited in its ability to handle complex text. Recent work by the authors involved automatically extracting car rental requirements from online text by using a domain knowledge base encoded in ontologies [26]. However, this method required a manually derived ontology to capture almost all domain terms, so it was not immediately suitable for general-purpose use.

## Methods

The goal of our method is to find existing rules that define the phenotype in a text snippet or to present closely related rules or rule templates that can be used to construct new concept definitions. Our method uses three techniques. First, we build a model of the rules and text snippets. Second, we use a similarity metric to compute the relatedness of rules to a snippet. Third, we find rules or rule templates that are the closest match to the text definition.

### Modeling rules and snippets as vectors

Our basic approach in the method is to compute the similarity between a snippet and the existing encoded rules for phenotype definitions. To perform this computation, we represent both the snippets and rules as vectors using a vector space model approach. Vector space modeling is widely used for information retrieval applications, since it provides an efficient and scalable computational approach for converting a text-based corpus to a standard mathematical format and then for searching for terms in that corpus.

#### ***Standard modeling of text snippets***

We use the standard vector space model to represent each snippet as a vector in Euclidian space, where each dimension of a vector corresponds to an individual term in the overall corpus of snippets. If a snippet includes a term, its value in the vector is given a non-zero weight for that term. We use the most common method to compute this weight, the term frequency-inverse document frequency (tf-idf) weighting. In this weighting scheme, weights increase proportionally to the number of the term appearances in the document but are scaled down by the frequency of the term in the corpus. The tf-idf formula used in this work is:

$$w_{i,d}=tf_{i,d}\times \log\left(n/df_{i}\right)$$

Where *w*_*i,d*_ is the tf-idf weight of term *i* in snippet*d*, *tf*_*i,d*_ is the frequency of term *i* in snippet*d*, *n* in the total number of snippets in the corpus, and *df*_*i*_ is the number of snippets that contain term *i*. Tf-idf weighs are computed for every term in a snippet. As an example we describe tf-idf weight computation for the term *language* from the sample snippet 1 in the Background section. The frequency of the term *language* in this snippet is 2. Assuming there are 10 snippets in our snippet corpus and 5 of them contain the term *language*, the tf-idf weight for this term is 2log2 ≈ 0.6. In the modeling process, tf-idf weights for all terms in a snippet are aggregated as the vector presentation of that snippet.

#### ***Semantic-based modeling of phenotypes***

A mathematical representation of the phenotype rules is also necessary to determine its closeness to a snippet. Again, we use the vector space modeling technique for this purpose. The terms for ontology classes and properties that are used in each rule are represented in the vector space. As described previously [27], we use the hierarchies of classes and properties in the ontology to extract indirectly related concepts and incorporate them in the vector representation. The weight of these related concepts is determined by using a semantic similarity metric, which exponentially decreases for each term based on its distance from the phenotype in the hierarchy graph. The formula for the semantic similarity is:

$$\text{Sim}\;\left(C_{1},C_{2}\right)=\;2^{‒\text{ShortestPath}}$$

Here, *Sim(C*_*1*_*, C*_*2*_*)* is the semantic similarity between concepts *C*_*1*_ and *C*_*2*_, and *ShortestPath* is the minimum distance between them in the class hierarchy graph. For example in our semantic-based modeling, the weight of the term *Words* in the vector representation of phenotype *Delayed Words* according to the class hierarchy in Figure 1 is 1.5. This is because the term *Words* has weight 1 in the phenotype’s name and weight 0.5 in the immediate parent’s name in the class hierarchy, based on the above semantic similarity formula.

#### ***Term-based modeling of phenotypes***

To evaluate whether semantic based modeling provides more relevant results in information extraction of phenotype definitions, we use an alternative approach for modeling phenotypes as vectors that is simply based on the terms used in the rule for that vector. If the rule contains a particular term, the value for that term is based simply on its frequency within the rule. The term-based approach does not include additional terms found in the class hierarchy and does not use semantic similarity measures as weights. As an example, the representation of *Delayed Words* phenotype in the term-based modeling is a vector with weights 1 for the terms *Delayed* and *Words*.

### Computing similarity

The information extraction task of identifying which phenotype concepts and/or related rule-based definitions may exist within a snippet can simply be done by measuring the similarity of vectors for existing rules within the encoded knowledge with the vector for a given snippet. Since both snippets and phenotype rules are modeled as vectors in a vector space, this similarity is calculated using the standard cosine similarity calculation. The cosine similarity for two vectors is the cosine of the angle between them, and ranges from 0 for orthogonal vectors to 1 for parallel vectors. The mathematical formula for cosine similarity is:

$$\text{Similarity}\left(a,b\right)=\text{cos}\left(\theta \right)=\left(a\cdot b\right)/\left(||a||\times ||b||\right)$$

Where *a* and *b* are two vectors in the Euclidean space, *θ* is the angle between them, *a · b* is their dot product, and ||a|| and ||b|| are the magnitudes of the vectors. Because cosine similarity is normalized by the sizes of the vectors, it is stable and independent of input vectors’ sizes and thus provides a robust measure of closeness.

### Evaluation and results

A key component of our experiment is to demonstrate whether including background knowledge improves the ranking of the correct rules, alternative rules, or rule templates for formalizing phenotype definitions in text. We thus compared our semantic-based method to the standard term-based method. As is common in text-mining, the term-based method uses the frequency of terms to model text and pre-existing rules as vectors. In this method the correlations between text sections and existing phenotype rule definitions is used to present the corresponding rules or rule templates to users to facilitate knowledge acquisition. In the semantic-based method related concepts to phenotypes are extracted from the class hierarchy in the domain knowledge base, and their relevance is quantified by a measure of semantic similarity. These relevance weights are incorporated in the vector representation of phenotypes, and similar to the term-based method, the correlation between text sections and existing rules is used to present the corresponding rules or rule templates for rule extraction. Using the domain ontology and the semantic similarity measure allow us to incorporate the domain context or *semantics* in our phenotype modeling, and is the basis for the naming of our method. In this work formalized phenotype definitions are equivalent to defining phenotype rules in the domain knowledge base, and we investigated the relevance of our resultsseparately in all possible scenarios.

### Autism phenotype knowledge base

We evaluated our method for the task of developing and maintaining a knowledge base in the domain of autism phenotyping. The initial autism phenotype knowledge base was composed of an ontology that was developed over a two-year period by a group of mental health experts, including the author AKD, and a knowledge-modeling expert [3]. The ontology contains both a class hierarchy that defines the terms and relationships among nine major categories of autism phenotypes such as language, social interaction, and behavioral abnormalities and a rule base that defines these concepts as value restrictions on research or clinical data collected through standardized diagnostic instruments. The scope of domain knowledge was initially defined by the experts who manually reviewed 26 relevant articles on autism phenotypes found in PubMed [3]. The experts then used OWL and SWRL to encode the phenotype definitions within the class hierarchy and rule base. The resulting knowledge base contains 1726 classes and properties and includes 156 SWRL rules that describe 145 unique phenotypic concepts for autism patients. In the process of knowledge acquisition, the domain experts identified the text sections within each paper that contained one or more autism phenotype definitions and associated the encoded rules to those definitions in each snippet. In our evaluation, these text sections are used as input snippets for our method, and the expert-confirmed associations between rules and snippets are considered to define the gold standard in each case. The annotations generated by the domain experts are not used in our method techniques and are only used for evaluation.

Our method outputs the most related rules in the existing knowledge base for each snippet, sorted by their cosine similarity; we have undertaken an evaluation of these results based on the three possible scenarios that we outlined in the Background section. We have focused our analysis on 53 phenotype concepts that use multiple criteria as part of their rule definition, providing the most difficult information extraction challenge. As is shown in the snippet examples 1 and 2 in the Background section, often several phenotypes are defined in a single snippet. Each snippet that is used in our evaluation contains several phenotype definitions. In total 10 snippets covered the definition of 53 phenotypes. The average length of these snippets is 98 terms and the standard deviation of their length is 42 terms.

### Scenario 1: existing rule definition

In the first scenario, the phenotype defined in the snippet is already present in the domain rule base. We evaluated this scenario by comparing the rank of rules for phenotypes that are known to exist within the snippet based on the domain experts mapping. For instance, assuming Delayed Words phenotype SWRL rule definition exists in the domain knowledge base, the snippet example 2 in the Background section is a snippet with an existing rule definition in the knowledge base. The gold standard in this scenario is to rank the existing rule definitions on the top of the result list. In our example Delayed Words phenotype SWRL rule definition should be ranked on the top.

We investigated this scenario by considering 53 domain expert-specified complex phenotype definitions in the autism phenotyping publications’ text. We then computed the average rank of correct phenotype rule in the returned sorted list of rule candidates as a measure of method’s accuracy. As can be seen in Figure 2, the average correct rank for the semantic-based method is better than the term-based method. The Wilcoxon signed-rank test shows that this difference is significant with p-value of 0.028. The average pairwise difference between correct phenotypes for these two methods is 1.15.

**Figure 3:**
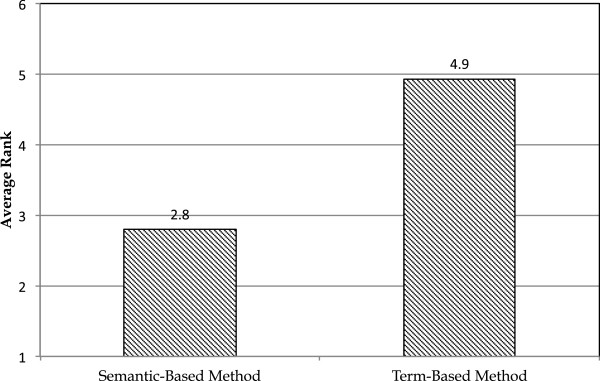
**Comparing semantic- and term-based methods in the second scenario.** Average ranks of the alternative phenotypes found in a snippet for the semantic- and term-based methods.

### Scenario 2: alternative rule definition

In this scenario, the exact definition of the phenotype concept present in the snippet does not exist in the rule base. Instead, the snippet defines a phenotype concept that has a different definition from the one encoded in existing rules for that concept. In this case, we undertook an evaluation by creating a set of different knowledge bases in which alternatively one of the existing rules for the concepts that have multiple rule definitions was removed. We then determined the rank of the alternative rules for the phenotype concept known to exist within a snippet. Delayed Phrases phenotype SWRL rule definition shows the rule definition of *Delayed Phrases* phenotype according the snippet example 2 in the Background section. As an example of this scenario, *Delayed Phrases* phenotype defined in the sample snippet 1 has a different criterion (age of first phrases ≥ 33–36 months) from Delayed Phrases phenotype SWRL rule definition (age of first phrases > 33 months). Assuming the corresponding rule for the phenotype defined in the sample snippet 1 does not exist in the rule base, the gold standard is to rank the alternative rule definitions on the top of the result list. In our example Delayed Phrases phenotype SWRL rule definition should be ranked on the top.

**Figure 4:**
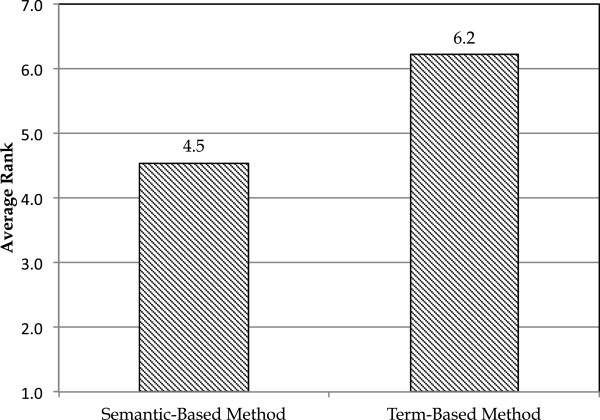
**Comparing semantic- and term-based methods in the third scenario.** Average ranks of the rule templates for a concept in a snippet for the semantic- and term-based methods.

The autism phenotype knowledge base contains 29 phenotype concepts with alternative rule definitions. To compare the semantic-based and the term-based methods, we computed the average rank for the correct results in this scenario. Figure 3 shows that the average rank of the correct answer in the semantic-based method is 2.8 and in the term-based method is 4.9. According to the Wilcoxon signed-rank test, p-value is 0.032. The average pairwise difference for ranks in these two methods is 2.8.

Delayed Phrases phenotype SWRL rule definition

ADI-R(?a) ^ adi-r2003:ADI_2003_acqorlossoflang_aphrase(?a, ?phraseage) ^ swrlb:greaterThan(?phraseage, 33) ^ adi_r2003:SubjectKey(?a, ?subjectID) ^ swrlx:createOWLThing(?phenotype, ?subjectID) → ‘Delayed phrases’(?phenotype) ^ autism-core:subject_has_quality_or_disposition(?subjectID, ?phenotype)

### Scenario 3: matching rule template

In this scenario, the snippet defines a phenotype that is not represented within the class hierarchy of the ontology and thus has no corresponding definition as a rule in the knowledge base. In this case, our method will still provide a set of ranked rules for concepts in the snippet, even though the actual concept does not exist in the ontology. We expect that the developer will recognize this situation by comparing the text with the returned results. We provide the developer the option of using a pre-defined template to enter the rule for the new concept. These templates derive from our prior work [28], in which we have found that SWRL rules can be represented by syntactic signatures based on the types of classes and properties they contain and their relationships. In a rule signature rules’ elements are represented by their types, and are grouped together if they are about a common subject. For example, if a rule defines a member from a class and assigns a property value to that member, the classes and property are grouped together as *(CD)* in the rule signature, where *C* represents the class and *D* represents the data value property. Full description of rule signatures requires close familiarity with SWRLsyntax [28], which is off-topic in this paper. However, in the case of the sample Delayed Words and Phrases phenotypes SWRL rule definitions, even an inexperienced user can observe the similarity between the rules’ structures and conclude that they have the same template. The syntactic analysis of several large SWRL rule bases indicated that they contained only a limited number of syntactic signatures, which we showed could be used as templates to acquire new rules. Our syntactic analysis of the autism phenotype rules found only 5 distinct signatures, and each of these mapped to semantically similar types of rule definitions [28].

We exploited this knowledge in our evaluation strategy for the third scenario. We created a set of 53 knowledge bases in which we alternatively removed one of the 53 distinct phenotype concepts and its corresponding rules. For each snippet that contained one of the concepts known to be missing in that version of the knowledge base, we compared the rank of the rule template for the returned rules with the correct rule template for the definition of the missing concept. As an example of this scenario, assuming *Delayed Words* phenotype does not exist in the domain ontology and rule base, the phenotype definition in the snippet examples 1 and 2 do not have the corresponding phenotype in the domain knowledge base. The gold standard in this scenario is to rank *Delayed Phrases* rule definition on the top of the result lists for these snippets as the rule with the similar template. Here, the goal is to provide phenotype rules that have a similar structure to the new phenotypes, so their syntactic templates can facilitate the acquisition of the new definitions.

We compared the average rank for the correct templates in this scenario, for the semantic and the term-based method. As can be seen in Figure 4, the average rank of the correct template is 4.5 in the semantic-based method and it is 6.2 in the term-based method. According to the Wilcoxon signed-rank test, the p-value for the comparison is 0.003. The average difference between correct template ranks between two methods is 1.7.

## Discussion

For researchers wishing to identify and encode phenotype definitions into formal knowledge, finding relevant articles and the related text fragments are only the first steps. The next—and most difficult—step is formally encoding the definitions contained in these text fragments. The current state-of-the art is simply to manually encode new definitions using the retrieved text as a guide. The ideal, of course, would be to automatically acquire the phenotype definition from text. As we showed in the Background section, the variety of ways phenotypes can be defined in free text makes this task very hard in practice. We thus have focused on developing a method that at least partially automates this authoring process and thus greatly assists users. This paper outlines the approach that we have developed to facilitate this knowledge acquisition process.

Our approach use semantic-based information retrieval techniques to help users both to identify known definitions of phenotypes in free text and to formalize the new definitions of phenotypes present in the text. Our work addresses the shortcomings of prior work where extracted knowledge is largely in the form of concept hierarchies. Biomedical knowledge about rule-based definitions, which are commonly used for criteria-based diagnoses, cannot be represented using such hierarchies. Our approach combines domain rule bases and ontologies with NLP techniques to capture this knowledge within vector space modeling. We have compared this approach with a more conventional term-based mechanism and our results have shown that incorporating domain knowledge into the information extraction method significantly improves the relevance of the results.

Our evaluation shows the text sections containing phenotype definitions often include several other phenotypes’ names and information without explicitly defining them. These occurrences in addition to the ambiguity and complexity of phenotype definitions in the form of free text complicate the rule extraction even in the case of existing phenotypes in the knowledge base. In the future, we are planning to improve the accuracy of our method by text understanding and natural language processing techniques. A limitation of the work is that we have only evaluated it in a single domain. We plan to further evaluate our approach in a variety of biomedical domains. In particular, we will evaluate the accuracy of our rule matching method with clinical guidelines. These sources typically contain a significant amount of proscriptive information, which is amenable to a rule-like representation. The ultimate goal is to produce a fully automated mechanism for finding and generating rules from text. By combining our information extraction approaches, we plan to create an overall workflow that starts with an existing ontology containing a set of rules, identify publication texts corresponding to new or related rules in the domain, and automatically extract new rules.

## Conclusions

The work described in this paper has demonstrated that the use of formally encoded domain knowledge can dramatically improve information extraction methods. Knowledge acquisition methods can also leverage this formal knowledge to provide a set of fully or partially automated strategies for generating new knowledge from text. Ultimately, these methods can help scientists to rapidly formalize the complex domain knowledge that is emerging in published research findings. They can also be applied to other information extraction challenges where there is a need to accurately capture computer-interpretable definitions, constraints, and policies that are specified in text.

## Competing interests

The authors declare that they have no competing interests.

## Authors’ contributions

All three authors were involved in the designing of the study, the analysis of the results, and drafting the manuscript. SH implemented the experiment and gathered the results. All authors read and approved the final manuscript.
